# Genomic landscape of *Mycobacterium tuberculosis*: Identifying mutation hotspots and stable regions for implications for drug development

**DOI:** 10.1016/j.nmni.2025.101674

**Published:** 2025-11-20

**Authors:** Muhammad Tahir Khan, Zeyu Luo, Arwa Omar Al Khatib, Dalal Sulaiman Alshaya, Ahmed A. Al-Qahtani, Tariq Nadeem

**Affiliations:** a State Key Laboratory of Respiratory Disease, Guangzhou Key Laboratory of Tuberculosis Research, Department of Clinical Laboratory, Guangzhou Chest Hospital, Institute of Tuberculosis, Guangzhou Medical University, Guangzhou, China; b Institute of Molecular Biology and Biotechnology, The University of Lahore, 58810, Pakistan; c School of Life Sciences, Chongqing Normal University, China; d Faculty of Pharmacy, Hourani Center for Applied Scientific Research, Al-Ahliyya Amman University, Amman, Jordan; e Department of Biology, College of Science, Princess Nourah bint Abdulrahman University, P.O. Box 84428, Riyadh, 11671, Saudi Arabia; f Department of Infection and Immunity, King Faisal Specialist Hospital and Research Center, Riyadh, 11211, Saudi Arabia; g Department of Microbiology, College of Medicine, Alfaisal University, Riyadh, Saudi Arabia; h Centre of Excellence in Molecular Biology, University of the Punjab, Lahore, 53700, Pakistan

**Keywords:** *Mycobacterium tuberculosis*, Genomes, Mutation frequencies

## Abstract

Whole-genome sequencing is the most promising approach for public health surveillance and antimicrobial drug resistance. The current study aimed to analyze base mutations across *Mycobacterium tuberculosis* genomes to assess mutation frequency and distribution across different regions. This study aimed to identify genomic regions of *Mycobacterium tuberculosis* with varying mutation frequencies to inform mechanisms of drug resistance and potential drug target discovery. The study analyzed base mutations across 209 whole genome sequences, which were aligned with reference H37Rv (NC_000962.3), using the PhyResSE pipeline. Based on the frequency of mutations, the regions have been classified into three main locations. High frequency mutation areas: around 2300 kb to 2400 kb, around 4100 kb to 4200 kb, around 1600 kb to 1700 kb, and 3700 kb to 3800 kb. These locations showed dense clusters linked to *katG* and *inhA* (Isoniazid resistance), Ethambutol resistance (*embB*), rifampicin resistance (compensatory role *rpoA).* Moderate frequency mutations were observed around 2000 kb to 2100 kb, around 1300 kb to 1400 kb, around 3800 kb to 3900 kb, and around 3400 kb to 3500 kb. These regions show mostly involved *rrs mutations* (amikacin, kanamycin, and capreomycin resistance), and *rpoA* has a compensatory role in rifampicin resistance. Regions exhibiting minimal mutation activity have been observed around 100 kb–300 kb, around 500 kb, and 2700 kb to 2800 kb. The most common nucleotide substitution was G to A (G→A) (16 %), followed by A to G (A→G) and T to C (T→C) (15 %). These findings collectively highlight novel genomic regions of stability and hypervariability, offering insights for refining WGS-based surveillance, resistance prediction, and future drug target prioritization.

## 1 Introduction

Tuberculosis (TB) is a fatal disease; each year, 1.3 million people die worldwide, and its causative agent, *Mycobacterium tuberculosis* (MTB), remains dormant in 90 % of TB-infected people (WHO 2023). The emergence of resistant MTB is a severe health issue and is a major obstacle to the global TB control program [1], [2]. The MTB, the causative agent of TB, primarily infects the lungs and may also spread to other body organs, called extrapulmonary TB [[3], [4], [5], [6]].

Given the global burden of TB, the rise of multidrug-resistant (MDR) and extensively drug-resistant (XDR) MTB exhibited critical scientific and clinical hurdles. A recent whole-genome study identified over 60 novel variants in isolates resistant to second-line, highlighting the evolving nature of the resistance landscape [7]. A more recent study found that approximately 5.7 % of drug-resistant TB exhibited resistance to Bedaquiline, which is a cornerstone of modern MDR-TB regimens [8].

Advances in WGS (whole-genome sequencing) offer a promising solution for public health and resistance mechanisms [[9], [10], [11], [12]]. It plays a pivotal role in novel drug design by providing insights into the genetic architecture, resistance mechanisms, and evolutionary dynamics of the pathogen, by identifying regions exhibiting minimal mutation activity, highly conserved, and essential genomic regions [12,13]. This approach has many advantages for public health surveillance and the molecular epidemiology of antimicrobial drug resistance, offering precise geographical tracking of pathogen transmission and enabling monitoring of pathogen incidence at the genotype level [14].

Despite ongoing control efforts, TB transmission persists due to latent infections, socio-economic differences, and limited access to diagnosis and treatment, particularly in high-burden countries. The global prevalence of MTB lineages shows distinct geographical patterns, such as Lineage 2 (East Asian/Beijing) and Lineage 4 (Euro-American), which are dominant in many regions and have been associated with increased transmissibility [15]. Understanding the population dynamics and lineage distribution is critical for public health interventions and tailored therapeutic approaches. Integration of WGS data with structural and functional annotation enables the identification of regions exhibiting minimal mutation activity, essential genomic areas suitable for structure-based drug design. Conversely, regions with high mutation density may show adaptive hotspots associated with drug resistance, immune evasion, or lineage-specific evolution. This genome-wide perspective thus integrates both evolutionary conservation and adaptive variability, providing a deeper understanding of MTB's genomic stability, mutational pressure, and drug target insights.

This study characterized the genome-wide distribution and frequency of base mutations across 209 MTB genomes to identify mutation hotspots and stable regions relevant to drug resistance and new drug target development.

## 2 Method details

### 2.1 Sequence data retrieval

The WGS data were selected based on drug resistance. The WGS data in SRA format (ERX-3360434-514, ERR2510337-445, ERR-2510546-66) in FASTQ format were retrieved from NCBI. These WGS data were deposited in GenBank under accession PRJE-B32684 and PRJEB-25972 (San Raffaele Scientific Institute) under the project First_10k_genomes_study_set_3, which were drug-resistant. WGS sequences and phenotypic resistance were performed on the first-line TB drugs on isolates from 16 countries on six continents under the CRyPTIC project ([16]).

### 2.2 Quality control and preprocessing

The retrieved WGS sequences were subjected to FASTQC for quality assessment to trim the low-quality raw reads. FastQC is a quality control check on SRA data retrieved from throughput sequencing machines. It ensures a set of analyses to give an impression of the data quality before further research. Sequencing reads that only met the defined quality criteria were retained for downstream analysis. Specifically, reads with an average Phred quality score below 30 or shorter than 50 bp were excluded. Adapter sequences and low-quality bases at the read ends were trimmed using Trimmomatic v0.39 [17].

### 2.3 Alignment and mutation analysis

The good-quality reads of genomes were compared with the MTB reference strain H37Rv (NC_000962.3) [18] using PhyResSE. The server is simple and more reliable for MTB WGS analysis. PhyResSE analyzes the Ion Torrent and NGS data in single and paired reads, applying QualiMap, FastQC, SAMtools, and BWA (Burrows-Wheeler Aligner) methods. The server performs comprehensive QC analysis, and mutational data from WGS were retrieved in CSV format and subjected to mutation statistical analysis and visualization [19]. Briefly, high-quality reads were aligned to the *Mycobacterium tuberculosis* H37Rv reference genome (NC_000962.3) using BWA-MEM v0.7.17. Variant calling and annotation were executed within the PhyResSE pipeline, filtering variants based on quality metrics. Variants with a minimum mapping quality <30, read depth <10 (allele frequency <75 %) were excluded. To prevent false-positive resistance calls, ambiguous variants with low base quality were automatically masked by the PhyResSE algorithm.

### 2.4 Statistical analysis and visualization

The study analyzed base mutations across 209 genomes of MTB samples to assess mutation frequency and distribution across the genome. This was visualized using a whole-genome Circos plot, illustrating mutation sites per base. Circos and chord diagrams provide an effective visual framework to display complex, large-scale genomic data. Circos plots illustrate the distribution and density of mutations across the *M. tuberculosis* genome, while chord diagrams highlight interconnections and co-occurrence patterns among resistance-associated genes, facilitating genomic relationships.

Relationships between mutation locations were further examined through chord diagrams depicting base mutations. Additionally, the frequency of different mutation types was quantified and presented in a pie chart. Statistical analysis was performed using the R programming language, version 4.1.2.

For generating the whole-genome Circos plot, the number of base mutations at each genomic site was calculated across all 209 genomes using the following formula:

(2.1) $${ALL}_{i}=\sum_{n=1}^{209}n_{i}$$

Where, ${ALL}_{i}$ total number of mutations observed at genomic site i across all 209 *Mycobacterium tuberculosis* isolates (genomic coordinate range: 0 to 4421400).

$n_{i}$ Denotes the number of mutations detected at site i in each sample (n = 1 to 209).

For generating the circos plot based on the statistical results, the study utilized the circlize package (R-4.1.2) described by Gu et al. (2014). The configuration of the circos plot involved setting parameters with circos. par (a): “gap.after” was set to 20° to space out sectors, “start.degree” was set to 87.5° to define the starting point of the visualization, and “gap.degree” was set to 3° to maintain clear separation between adjacent genomic sectors. Additionally, a cytoband text file was employed to define chromosome segments. For example, the segment labeled “All Sample” spanned from position 0 to 4,421,400, designated as chromosome "'All sample'", with additional information specified as “n/a" [20].

To draw base mutation chord diagrams, the genomic mutation data were first processed by cleaning up missing values and categorizing mutations into types such as A→T, A→G, A→C, T→A, T→G, T→C, G→A, G→T, G→C, C→A, C→T, and C→G. These types represent mutations where the first base is the original (lost) base and the second is the new (gained) base. The data were then segmented into different types of groups across 209 samples. The circlize package (version 0.4.15) was utilized to facilitate this processing and draw the chord diagrams based on relationships between gained and lost mutations.

To illustrate the distribution of these mutation types across the samples, a mutation percentage pie plot was created. This plot shows the statistical percentage of each type of base mutation. The mathematical formula used to calculate the percentage of each mutation type is as follows:

(2.2) $$Percentage\;of\;Mutation\;Type=\frac{\;Number\;of\;Specific\;Mutation\;Type}{Total\;Number\;of\;Mutation}$$

where “Mutation Type” refers to A→T, A→G, etc.

The first step is to create 3 folders: data, output2, cytoband, and 209 original mutation data files were kept in data. The second step is to execute code: step 1 read the original data and step 2 calculates base mutations in the 209 samples, and draws All sample circos plots, scatter plots, frequency distribution maps, and percentage maps. Step 3 led the substitution of base mutations in each single sample base mutations and a percentage map was plotted. Every outcome was automatically saved in the output2 folder.

## 3 Results

### 3.1 Mutation frequency and genomic distribution

**Fig. 1:**
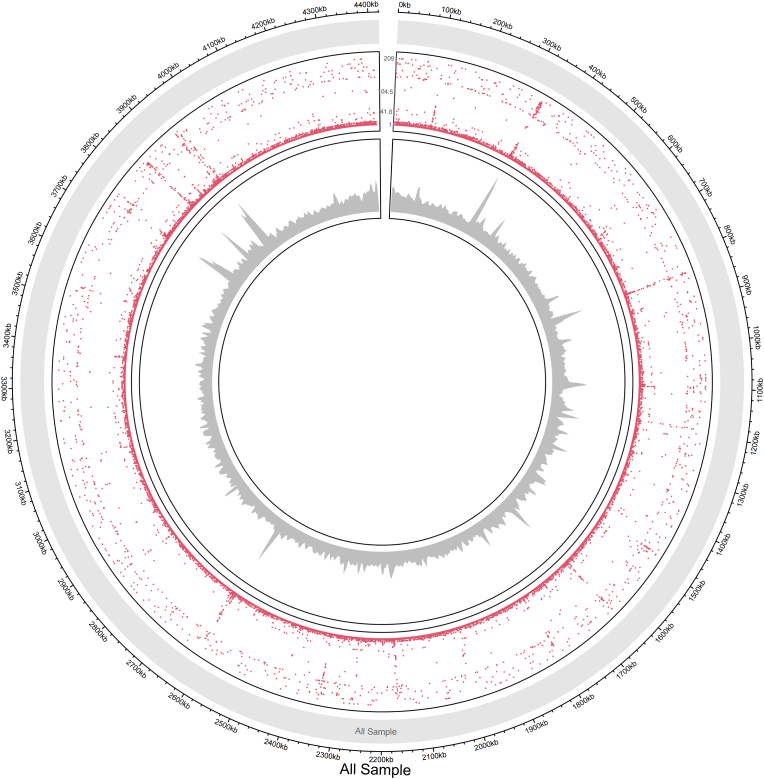
Genome-wide mutation-density Circos. Circos plot of 209 whole-genomes. The height of the red spots in the outer circle indicates the number of sites per mutation, while the gray band in the inner circle is the genomic density map. The outer circle of the plot with red spots indicates mutation sites, with high-frequency mutations, taller and more densely packed red spots.

Mutation frequencies were recorded across the genome of MTB, covering a range from 1 kilobase (kb) to 4400 kb in 209 genomic isolates. The plot in Fig. 1 shows distinct regions with varying mutation frequencies. Genomic regions were classified as high mutation frequency locations, moderate mutation frequency locations, and regions exhibiting minimal mutation activity.

High mutation frequencies were observed in several areas: the top right quadrant (around 2300 kb to 2400 kb), the top left quadrant around 4100 kb to 4200 kb, the bottom right quadrant around 1600 kb to 1700 kb, and the bottom left quadrant around 3700 kb to 3800 kb. These locations showed dense clusters (Fig. 1) and linked *katG* and *inhA* (Isoniazid resistance), ethambutol resistance (*embB*), rifampicin resistance (with compensatory role *rpoA).* High mutation densities observed in these genes correspond to known drug resistance loci and these regions are subject to positive selection because they provide a strong survival advantage under antibiotic stress.

Moderate mutation frequencies were noted in the top right quadrant around 2000 kb to 2100 kb, the bottom right quadrant around 1300 kb to 1400 kb, the top left quadrant (around 3800 kb to 3900 kb), and the bottom left quadrant (around 3400 kb to 3500 kb). Isoniazid resistance (*katG* mutations), amikacin, kanamycin, and capreomycin resistance (*rrs* mutations), and *rpoA* mutation have a compensatory role in rifampicin resistance. Regions exhibiting minimal mutation activity have been observed in the center regions, the top right quadrant (around 100 kb–300 kb), and the bottom right quadrant (around 2700 kb to 2800 kb) (Fig. 1).

Furthermore, regions with no mutations might indicate stable genomic segments less susceptible to mutations. For example, specific segments between 10 kb and 100 kb, where very few red spots (Fig. 1) can be identified as regions exhibiting minimal mutation activity. Identifying multidrug-resistant (MDR) isolates with stable genomic segments for potential drug design is an important approach for better management in the future.

**Fig. 2:**
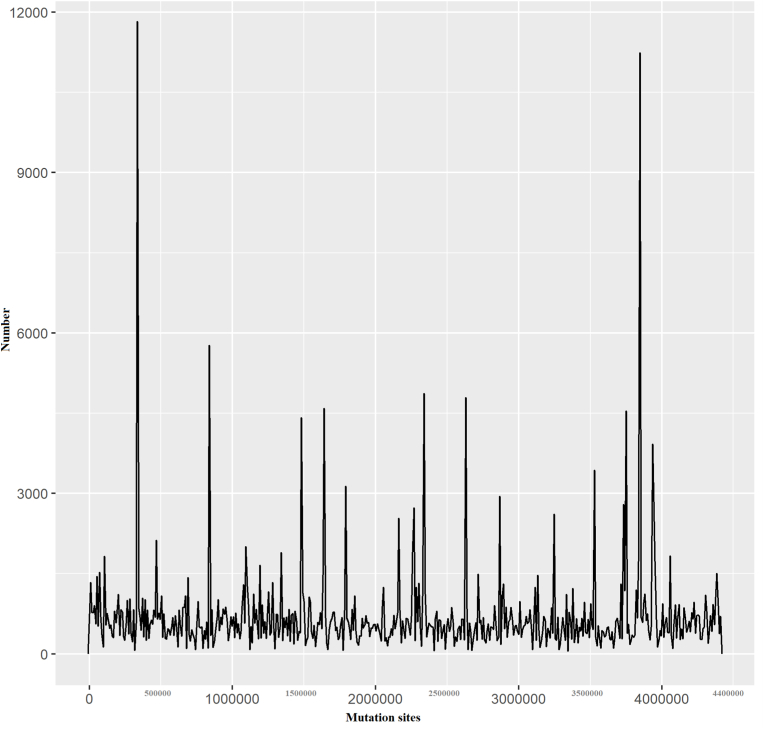
Frequency and mutation sites along the genome Frequency distribution maps for 209 samples, with each histogram bin range set at 8822, differ from the genomic density settings in Fig. 1. The x-axis represents mutation sites along the genome, while the y-axis indicates the number of mutations observed at each site. This depiction allows for an in-depth analysis of regions with high, moderate, and low mutation frequencies.

The comprehensive visualization of mutation distribution across various genomic regions has been shown (Fig. 2). The peaks with a highest number of mutations are: Peak 1 at around 400 kb, with a mutation frequency of approximately 11800. This could be a highly mutable region, possibly under intense selective pressure. The second high Peak 2 around 800 kb, with a frequency of about 5800 mutations. The region has two important targets of RIF *rpoB, RpoC* (location 759.807–763.325 kb, 763.370–767.320), and STR; *rpsL* (location 781.560–781.934 kb) (See Table 1). The third peak at 3800 kb harbored about 11000 mutations. Other peaks are scattered throughout the region, with frequencies ranging from 3000 to 5500 mutations.

**Table 1:** Genes involved in drug resistance and their genomic location.

Drug	Gene involved in resistance	Location (kb)
INH	*katG*	2153.889–2156.111
INH	*inhA*	1674.202–1675.011
RIF	*rpoB*	759.807–763.325
	*rpoA*	3877.464–3878.507
	*rpoC*	763.370–767.320
PZA	*pncA*	2288.681–2289.241
PZA	*panD*	4043.862–4044.281
EMB	*embB*	4246.514–4249.810
STR	*rpsL*	781.560–781.934
AMK, KAN, CAP	*rrs*	1471.846–1473.382
FQs	*gyrA*	7.302–9.818
FQs	*gyrB*	5.240–7.267

Isoniazid: INH, Rifampicin: RIF, Ethambutol: EMB, Streptomycin: STR, Amikacin: AMK, Kanamycin: KAN, and Capreomycin: CAP, Fluoroquinolones: FQs.

Firstly, distinct regions in the genome have exceptionally high mutation frequencies. At approximately 400 kb (around *gyrA* and *gyrB* regions involved in fluoroquinolone resistance), a prominent peak with around 11,800 mutations indicates a significant mutation hotspot. These peaks highlight critical areas of the genome where mutations are highly concentrated, suggesting potential regions of significant genetic alterations.

In addition to these hotspots, several regions had moderate mutation frequencies. Notable peaks can be seen, where the number of mutations reaches about 3000–4000. Other moderate peaks have been observed near 1500 kb and 2300 kb, with mutation counts fluctuating between 2000 and 4600 (*rpoB*: Rifampicin resistance, *rpsL:* Streptomycin resistance). These areas suggest regions of the genome that, while not as intensely mutated as the hotspots, still experience considerable genetic alterations. The region above 4100–4400 kb also has low-frequency mutations.

There are some regions with relatively low mutation frequencies. These areas are characterized by mutation counts remaining below 1000 and are dispersed throughout the genome, interspersed between higher peaks (Fig. 2). These low-mutation regions indicate segments of the genome that are more stable and less prone to genetic changes, highlighting the inherent variability in mutation distribution across different genomic regions.

### 3.2 Nucleotide substitution patterns

**Fig. 3:**
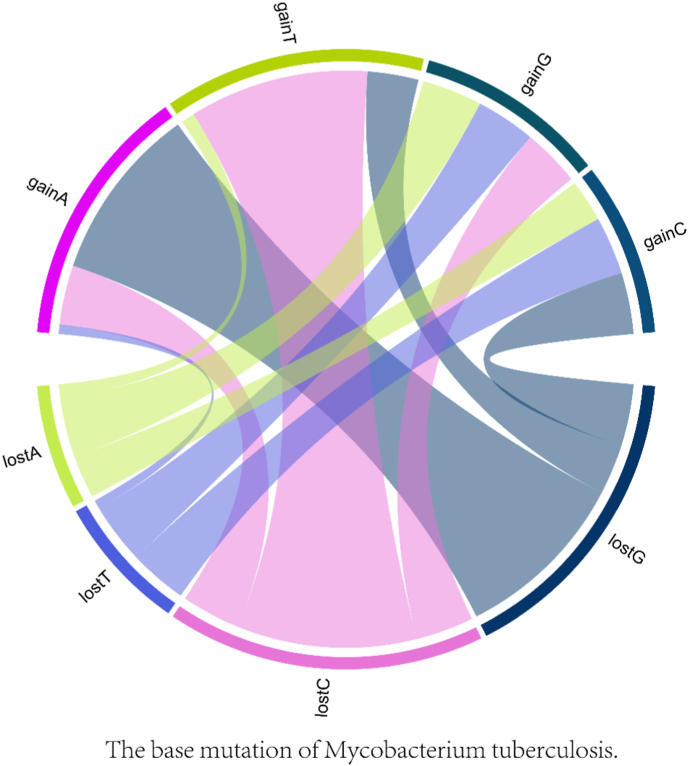
Loss and gain mutations of nucleotides in MTB genomes Chord diagrams of loss and gain mutations of nucleotides in MTB genomes. Each segment on the outer circle represents these nucleotide bases, divided into gain and loss groups. The gain of adenine (gainA) while its loss A (lostA). The thickness of these bands indicates the frequency or intensity of these mutations, with thicker bands signifying a higher frequency of nucleotide changes.

Focusing on specific nucleotide patterns, Fig. 3 reveals that gainA is frequently associated with lostG and lostT. This is evident from the substantial connectivity between the “gainA” and the “lostG” (blue) and “lostT” (pink) segments. Loss of adenine is often accompanied by gain of guanine or cytosine (C), as shown by the connections from “lostA” (green) to “gainG” (green) and “gainC” (blue) (Fig. 3).

Thymine (T) mutations show that gainT in are common when adenine and cytosine are lost (lostA” (green) and “lostC” (blue)) (See Fig. 3). The lostT frequently leads to gainG and “C”, as indicated by the connections from “lostT” (blue) to “gainG” (dark blue) and “gainC” (blue). The gainG (dark blue), is significantly linked to the loss of adenine and thymine. This pattern is obvious from lostA and lostT. The lostG results in gains of adenine and cytosine i.e. “gainA” (magenta) and “gainC” (blue).

If we observe the cytosine (C), the gain patterns showed that C is often gained when T and G are lost. These can be seen from “gainC” (blue) to “lostT” (blue) and “lostG” (blue). The lostC typically results in gains of A and T, where gainA (magenta) and gainT (lime green) (Fig. 3).

**Fig. 4:**
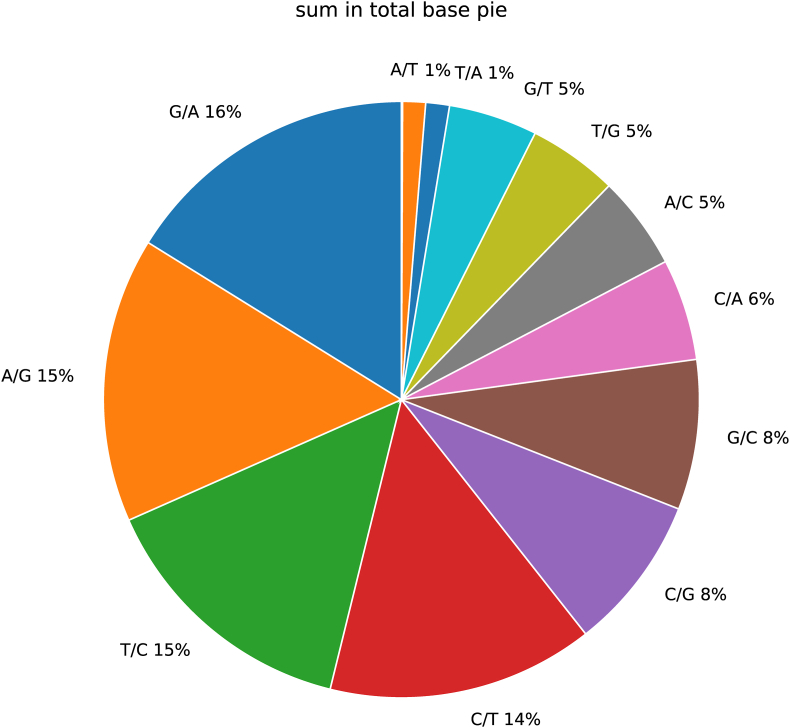
Nucleotide substitution (%) pie plot of 209 genomes samples "other” category accounts for 0 % of the total, indicating that substitutions not listed specifically in the chart are negligible or nonexistent in the dataset.

The most common nucleotide substitution was G to A (G→A), which accounts for 16 % of the total substitutions (Fig. 4). The A to G (A→G) and T to C (T→C) substitutions are closely followed, each making up 15 % of the total. These substitutions indicate a high frequency of transitions between these nucleotides in the MTB genomes. Next in frequency are the C to T (C→T) substitutions (14 %) and C to G (C→G) and G to C (G→C) substitutions (8 % each). These substitutions highlight the variability in the cytosine and guanine bases within the genome.

The substitutions A to C (A→C) and T to G (T→G) both account for 5 % of the total, indicating moderate levels of these transversions. Similarly, G to T (G→T) substitutions also make up 5 % of the total substitutions, showing a similar frequency to A→C and T→G substitutions. Less frequent substitutions include C to A (C→A) (6 %) and A to T (A→T), and T to A (T→A), each at 1 %. These low-frequency substitutions suggest that these transversions are less common in the MTB genomes.

The most frequent nucleotide substitutions in MTB genomes are transitions, particularly G to A and A to G, along with T to C and C to T (Fig. 4). Transversions, such as C to G, G to C, A to C, T to G, and G to T, occur less frequently, with the least common substitutions being A to T and T to A. Understanding these substitution patterns is crucial for studying the mutation mechanisms, the evolutionary dynamics of MTB genomes, and the cause of MDR.

### 3.3 Functional genomic distribution (coding vs. non-coding)

**Fig. 5:**
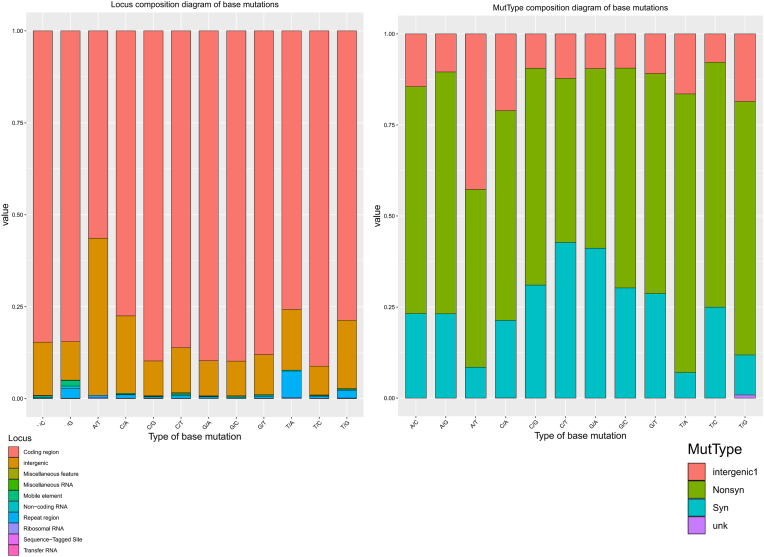
Locus composition of mutation in the genome regions The graphs display the locus composition and mutation type composition of base mutations in MTB genomes. The left bar chart represents the locus composition diagram of base mutations. The chart categorizes base mutations based on their genomic location. The right bar chart depicts the mutation type composition of base mutations, categorizing mutations based on their type.

As shown in Fig. 5, the coding region (red) is the predominant site for mutations across all base types (A→C, A→G, A→T, C→A, C→G, C→T, G→A, G→C, G→T, T→A, T→C, T→G). This indicates that coding regions are the primary sites for nucleotide changes, potentially affecting protein function and drug-target interaction, leading to MDR. Intergenic regions (pink) also exhibit a significant proportion of mutations, although less frequently than in coding regions. Mutations in miscellaneous features (brown) are particularly in specific mutations like G→T and C→A. Non-coding RNA regions (orange) and ribosomal RNA regions (blue) have fewer mutations. Non-coding RNA has a slightly higher frequency in mutations like G→T and C→A. Repeat regions (dark blue) and transfer RNA (dark blue) show minimal mutation occurrences. Miscellaneous RNA (light blue) and mobile elements (light blue) have sparse mutations. The sequencer-tagged site (STS) category (black) shows the least occurrence of mutations across all types.

Intergenic mutations (Fig. 5) are prevalent across all base types, indicating that non-coding regions between genes also undergo significant changes. Nonsynonymous mutations constitute a substantial portion of the mutations, leading to amino acid changes in proteins and potentially altering protein function.

### 3.4 Gene-specific resistance mutations

As shown in Table 2, among the top drug resistance genes, *rpoB* shows the highest number of mutations overall (585), with 191 mutations directly implicated in resistance and 323 accounted in genotyping. This highlights its critical role in RIF resistance and its frequent use as a marker in molecular diagnostics. The *gyrA + gyrB* also exhibits a significant number of mutations (794 total), with 109 contributing to FQs resistance, and 228 are used in genotyping (See Supplementary File S1).

**Table 2:** Mutations frequencies in drug target genes. These are involved in resistance among 209 MTB genomic isolates.

Gene involved in resistance	No. of mutations	Detection of resistance	Detection in genotyping	Unknown/other
*katG*	366	160	170	36
*inhA*	9	3	–	6
*rpoB*	*585*	191	323	71
*rpoA*	*18*	–	–	18
*RpoC*	*124*	–	35	89
*pncA*	*194*	59	111	24
*panD*	*3*	–	–	3
*embB*	272	149	11	112
*rpsL*	70	68	–	2
*rrs*	281	29	–	252
*gyrA + gyrB*	794	109	228	457

For INH resistance, *katG* and *inhA* are key genes. *katG* has a large number of mutations (366 total), with 160 directly involved in resistance and 170 in genotyping. Although *inhA* has fewer mutations (No. 9), three of which are associated with resistance.

Other genes contributing to drug resistance include *embB* (149 out of 272 total mutations), primarily associated with ethambutol resistance, and rpsL (68 out of 70 total mutations), which has been linked to STR resistance.

Some genes, such as rpoA and RpoC, appear to have fewer mutations directly linked to resistance in this dataset. In *rpoA,* has 18 total mutations in the “unknown/other” category. Similarly, RpoC has 124 mutations, with 35 as genotyping and a large number (89) falling into the “unknown/other” group. In rrs, despite having 281 total mutations, only 29 were involved in resistance, with 252 categorized as “unknown/other,” suggesting a more complex or less well-defined role in resistance mechanisms, or may be the involvement in resistance to other drugs.

In summary, the identified high-frequency regions (2300–2400 kb, 4100–4200 kb, 1600–1700 kb, and 3700–3800 kb) coincide with known drug resistance loci (*katG, inhA, embB, and rpoA*) while mutation-sparse regions (10–100 kb and 2700–2800 kb) may represent conserved. The observed transition bias G→A (16 %), A→G, and T→C (15 %) is differs in frequency from previously reported global MTB datasets. These findings collectively highlight novel genomic regions of stability and hypervariability, offering insights for refining WGS-based surveillance, resistance prediction, and future drug target prioritization.

## 4 Discussion

The high prevalence of mutations in genes associated with drug resistance, particularly *rpoB, katG*, and *embB* in MTB isolates, is a significant concern for public health. The dynamic interaction of nucleotide mutations in MTB genomes has been shown (Fig. 3), with significant cyclical patterns where nucleotides are frequently gained and lost [21,22]. The complexity and frequency of these mutations showcase how one nucleotide's gain often correlates with another's loss. This information could be crucial for understanding the mutation mechanisms in MTB and potentially for developing targeted interventions and understanding drug resistance.

The regions exhibiting minimal mutation activity are purifying selection, indicating that sequence variation in these loci is poorly tolerated and therefore rapidly removed from clinical populations [23,24]. Experimental transposon-sequencing (Tn-seq) demonstrates that many conserved regions overlap experimentally validated essential genes, providing direct evidence that disruption may reduce fitness [23]. The recognized drug-resistance hotspots showed repeated, convergent mutations due to positive selection [13], highlighting the different selective regimes acting on mutable resistance loci versus mutation-intolerant targets.

The high mutation frequency regions overlap with genomic areas enriched in PE/PPE family genes and drug resistance determinants (*katG, inhA, embB, and rpoA*). *PE/PPE* are known to be highly variable, associated with antigenic variation and host-pathogen interactions, contributing to immune evasion [18,25].

The regions exhibiting minimal mutation activity, observed, likely represent functionally essential and evolutionarily conserved loci that are under strong negative selection [24]. Because mutations in these loci can lead to loss of function or reduced fitness, they are selectively eliminated from the population, resulting in highly stable, mutation-intolerant regions across diverse clinical isolates [24]. Taken together, these low-frequency mutational loci are attractive drug targets because resistance-conferring substitutions would incur a high fitness cost. For a drug-development pipeline, we recommend (i) cross-referencing each region exhibiting minimal mutation activity, (ii) prioritizing essential proteins with structural tractability for structure-based inhibitor design.

The most frequent nucleotide substitutions in MTB genomes are transitions (Fig. 4), which play a significant role in the development of drug resistance. While substitutions may confer drug resistance, they often come with a fitness cost [26]. However, compensatory mutations can alleviate these costs. The transversions occur less frequently, with the least common substitutions being A to T and T to A. Understanding these substitution patterns is crucial for studying the mutation mechanisms and evolutionary dynamics of MTB genomes. In the MTB genomes, transitions are the most frequent type of nucleotide substitutions. The G→C > A→T transitions occurred more frequently than A→T > G→C transitions [27], indicating a significant bias to transitions. This transition bias is consistent with genome-wide mutation patterns observed in antibiotic resistance-associated mutations [27]. Another study reported single nucleotide variants (SNVs) in MTB isolates resistant to various antibiotics revealed that A > G mutations were the most predominant (14 % of isolates resistant to ofloxacin, moxifloxacin, and amikacin) [28]. The high frequency of transitions can be attributed to the intrinsic factors of DNA polymerases and also the selective pressures of the host immune system [27]. Transversions are less frequent than transitions in the genomes of MTB. However, they still play a significant role in the evolution and drug resistance. A single transversion accounted for over 70 % of the mutational events conferring isoniazid resistance [27].

The base mutations in MTB genomes predominantly occur in coding and intergenic regions (Fig. 5). The prevalence of mutations in coding regions suggests that the genomic changes substantially impact gene function and regulation [29,30]. Mutations in intergenic regions highlight the evolutionary changes occurring in non-coding parts of the genome [31,32]. The current study data indicate a dynamic mutation landscape in MTB genomes, affecting both coding and non-coding regions with significant functional implications.

The mutation densities in MTB genomes near 10 kb and 40 kb appear high, indicating that these regions could be more susceptible to mutations due to their functional importance or structural characteristics [33]. Drug resistance due to genetic mutation may be under selective pressure, leading to higher mutation rates [34]. High mutation densities in *katG, inhA*, *rpoB*, and *embB* are subject to positive selection because they provide a strong survival advantage under antibiotic stress [35]. Low-mutation or regions exhibiting minimal mutation activity are often associated with essential genes involved in cellular functions (replication, transcription, or metabolism). The evolutionary conservation of these regions suggests strong purifying selection [23].

Some studies reported that a 20 kb variable region is a hotspot for genetic events, which often contains the IS6110 insertion sequences and facilitates homologous recombination and deletions. These deletions are associated with the presence of IS6110 and contribute to the genomic diversity among MTB clinical isolates [36,37].

At approximately 400 kb, a prominent peak with around 11,800 mutations indicates a mutation hotspot. A previous study of 1960 MTB clinical isolates revealed some regions in the MTB genome showing a high density of mutations, often associated with drug resistance [33]. These mutational hotspot regions may be under selective pressure. Moreover, the presence of repetitive DNA sequences or transposable elements in these regions may also contribute to the high frequency of mutation rates [38]. Understanding these mutation hotspots is crucial for developing effective strategies to combat TB, drug-resistant strains [39].

The current study also observed that certain regions in the MTB genome exhibit relatively low mutation frequencies (See Fig. 2, Fig. 5) and are dispersed throughout the genome. These findings show the inherent variability in mutation distribution across different genomic regions. These low-mutation regions, often called cold spots, are interspersed and indicate more stable and less prone to genetic changes [28].

The failure of therapeutic approaches may be linked to nucleotide connectivity patterns. In our study, Fig. 3 reveals that gainA mutations are associated with lostG and lostT. Similarly, this important connectivity between the “gainA” and the “lostG” (blue) and “lostT” (pink) segments can also be observed. The lostA is often substituted with G or C, “lostA” (green) to “gainG” (green) and “gainC” (blue). This pattern suggests that specific nucleotide changes are more prevalent and may be interconnected in the emergence of drug resistance. This is evident from the mutations in the *rpoB* gene, which are associated with rifampin resistance and often involve A-to-G transitions [38]. The high frequency of G→A and A→G transitions is particularly associated with specific drug resistance mechanisms in MTB [40]. Similarly, mutations in the *katG* gene, linked to isoniazid resistance, often involve A→G transitions. These findings highlight the importance of understanding the specific nucleotide substitution for novel therapeutic approaches [41]. Recent WGS investigations of MTB clinical isolates have documented transition-bias and region-specific mutation densities. For instance, a study of 716 XDR-MTB isolates, investigators detected significant enrichment of transition (A↔G, C↔T) mutations within hotspot genes under drug pressure [28].

A recent meta-analysis of clinical genomic isolates estimated a molecular clock rate below 1 SNP per genome per year, showing evolutionary stability in many segments [42]. These findings align with our detection of genomic regions with very low mutation frequency, supporting the view that such zones are under strong purifying selection and may constitute stable targets for drug design.

The coding region is the predominant site for mutations (See Fig. 5) across all base types (A→C, A→G, A→T, C→A, C→G, C→T, G→A, G→C, G→T, T→A, T→C, T→G) in drug-resistant MTB genomes, showing that these are the primary sites for changes. Mutations in coding regions can directly affect protein function and drug-target interactions, leading to MDR [43].

The transitions of T→C and C→T may have a role in the evolution and adaptability of MTB, which can also lead to changes in gene expression, affecting bacteria's drug resistance. The relatively lower frequency of C→G and G→C substitutions may be less common, but may contribute to the overall genetic diversity. The observed mutation patterns provide insights into selective pressures, including antibiotics, that drive adaptive evolution in MTB, mainly in resistance-associated genes (*rpoB, katG, and gyrA*) [44]. Identifying high and low frequencies of mutational regions enables the difference between adaptive mutations involved in drug resistance and conserved, low-frequency mutation loci required for bacterial survival [23]. This has direct implications for surveillance, allowing detection of emerging resistance mutations, and for therapeutic use, as conserved regions serve as promising targets.

The study revealed that high-resolution maps of mutation density allow precision genomic surveillance from conserved and mutation-intolerant segments. Integrating these density maps into WGS surveillance improves detection sensitivity for emerging drug resistance: hotspot regions can be prioritized for real-time monitoring and targeted sequencing panels. This targeted approach will increase diagnostic performance for better management from limited sequencing throughput, an important advantage for high-burden countries. For drug-susceptibility testing and diagnostic strategy, mutation-density profiles will be helpful to distinguish where rapid molecular tests should focus and where WGS is warranted. High-density, rapidly evolving loci justify targeted NGS assays for rapid DST calls.

### 4.1 Limitations of the study

Although distinct and low-variation regions are evident, this limited dataset (n = 209) may not fully capture the genetic diversity of MTB, and therefore, the apparent “Regions exhibiting minimal mutation activity” zones should be considered provisional rather than absolute, as additional sampling could reveal further variability across these genomic regions.

## 5 Conclusion

MTB genomes reveal distinct patterns and frequencies of nucleotide substitutions across different genomic regions. High mutation frequencies were observed in around 2300 kb to 2400 kb, around 4100 kb to 4200 kb, 1600 kb to 1700 kb, and around 3700 kb to 3800 kb. Moderate mutation frequencies were observed around 2000 kb to 2100 kb, around 1300 kb to 1400 kb, around 3800 kb to 3900 kb, and around 3400 kb to 3500 kb. Regions exhibiting minimal mutation activity have been observed around 100 kb–300 kb, and around 2700 kb to 2800 kb. The most common nucleotide substitution is G to A, accounting for 16 % of total substitutions, followed by A to G and T to C (15 %). Transversions occur less frequently, with the least common substitutions being A to T and T to A. The study reveals a diverse landscape of mutations, with rpoB, katG, and embB emerging as major contributors. This has direct implications for surveillance and for therapeutic as conserved regions serve as promising targets. The high-resolution maps of mutation density allow precision genomic surveillance. Integrating these density maps into WGS surveillance can be prioritized for real-time monitoring and targeted sequencing panels. This targeted approach will increase diagnostic performance, an important advantage for high-burden countries. High-density, rapidly evolving loci justify targeted NGS assays for rapid DST calls. These findings collectively highlight novel genomic regions of stability and hypervariability, offering insights for refining WGS-based surveillance, resistance prediction, and future drug target prioritization.

## CRediT authorship contribution statement

**Muhammad Tahir Khan:** Writing – original draft, Formal analysis, Conceptualization. **Zeyu Luo:** Methodology, Data curation. **Arwa Omar Al Khatib:** Resources, Funding acquisition. **Dalal Sulaiman Alshaya:** Funding acquisition, Formal analysis. **Ahmed A. Al-Qahtani:** Resources, Funding acquisition, Data curation. **Tariq Nadeem:** Writing – review & editing, Data curation.

## Availability of data

The genomic data in the current study could be accessed at GenBank Accession Numbers ERR2510337-ERR2510445, ERR2510546-ERR2510645. Supplementary Data contains all mutations in 209 genomes and could be provided on reasonable request to the corresponding authors as Supplementary File S1. Code and other supplementary data are available as S2.

## Ethics statements

NA.

## Declaration of competing interest

The authors declare that they have no known competing financial interests or personal relationships that could have appeared to influence the work reported in this paper.

## References

[1] WHO. WHO Global tuberculosis report (2019). http://www.who.int/tb/publications/global_report/en/.

[2] Li S., Tan Y., Deng Y., Bai G., Huang M., Shang Y. (2024). The emerging threat of fluroquinolone-, bedaquiline-, and linezolid-resistant Mycobacterium tuberculosis in China: observations on surveillance data. J Infect Public Health.

[3] Allahyartorkaman M., Mirsaeidi M., Hamzehloo G., Amini S., Zakiloo M., Nasiri M.J. (2019). Low diagnostic accuracy of xpert MTB/RIF assay for extrapulmonary tuberculosis: a multicenter surveillance. Sci Rep.

[4] Gopalaswamy R., Dusthackeer V.N.A., Kannayan S., Subbian S. (2021). Extrapulmonary tuberculosis—an update on the diagnosis, treatment and drug resistance. J Respir.

[5] Mtetwa H.N., Amoah I.D., Kumari S., Bux F., Reddy P. (2022). The source and fate of Mycobacterium tuberculosis complex in wastewater and possible routes of transmission. BMC Public Health.

[6] Chandra P., Grigsby S.J., Philips J.A. (2022). Immune evasion and provocation by Mycobacterium tuberculosis. Nat Rev Microbiol.

[7] Qian W., Ma N., Zeng X., Shi M., Wang M., Yang Z. (2024). Identification of novel single nucleotide variants in the drug resistance mechanism of Mycobacterium tuberculosis isolates by whole-genome analysis. BMC Genom.

[8] Hu X., Wu Z., Lei J., Zhu Y., Gao J. (2025). Prevalence of bedaquiline resistance in patients with drug-resistant tuberculosis: a systematic review and meta-analysis. BMC Infect Dis.

[9] Bagger F.O., Borgwardt L., Jespersen A.S., Hansen A.R., Bertelsen B., Kodama M. (2024). Whole genome sequencing in clinical practice. BMC Med Genom.

[10] Li J., Yang T., Hong C., Yang Z., Wu L., Gao Q. (2022). Whole-genome sequencing for resistance level prediction in multidrug-resistant tuberculosis. Microbiol Spectr.

[11] Pankhurst L.J., Del Ojo Elias C., Votintseva A.A., Walker T.M., Cole K., Davies J. (2016). Rapid, comprehensive, and affordable mycobacterial diagnosis with whole-genome sequencing: a prospective study. Lancet Respir Med.

[12] Walker T.M., Kohl T.A., Omar S.V., Hedge J., Del Ojo Elias C., Bradley P. (2015). Whole-genome sequencing for prediction of Mycobacterium tuberculosis drug susceptibility and resistance: a retrospective cohort study. Lancet Infect Dis.

[13] Farhat M.R., Shapiro B.J., Kieser K.J., Sultana R., Jacobson K.R., Victor T.C. (2013). Genomic analysis identifies targets of convergent positive selection in drug resistant Mycobacterium tuberculosis. Nat Genet.

[14] Revez J., Espinosa L., Albiger B., Leitmeyer K.C., Struelens M.J., ECDC National Microbiology Focal Points and Experts Group (2017). Survey on the use of whole-genome sequencing for infectious diseases surveillance: rapid expansion of European national capacities, 2015–2016. Front Public Health.

[15] Freschi L., Vargas R., Husain A., Kamal S.M.M., Skrahina A., Tahseen S. (2021). Population structure, biogeography and transmissibility of Mycobacterium tuberculosis. Nat Commun.

[16] Allix-Béguec C., Arandjelovic I., Bi L., Beckert P., Bonnet M., CRyPTIC Consortium and the 100,000 Genomes Project (2018). Prediction of susceptibility to first-line tuberculosis drugs by DNA sequencing. N Engl J Med.

[17] Bolger A.M., Lohse M., Usadel B. (2014). Trimmomatic: a flexible trimmer for illumina sequence data. Bioinformatics.

[18] Cole S.T., Brosch R., Parkhill J., Garnier T., Churcher C., Harris D. (1998). Deciphering the biology of Mycobacterium tuberculosis from the complete genome sequence. Nature.

[19] Feuerriegel S., Schleusener V., Beckert P., Kohl T.A., Miotto P., Cirillo D.M. (2015). PhyResSE: a web tool delineating Mycobacterium tuberculosis antibiotic resistance and lineage from whole-genome sequencing data. J Clin Microbiol.

[20] Gu Z., Gu L., Eils R., Schlesner M., Brors B. (2014). Circlize implements and enhances circular visualization in R. Bioinformatics.

[21] Fleischmann R.D., Alland D., Eisen J.A., Carpenter L., White O., Peterson J. (2002). Whole-genome comparison of Mycobacterium tuberculosis clinical and laboratory strains. J Bacteriol.

[22] Pelly S., Bishai W.R., Lamichhane G. (2012). A screen for non-coding RNA in Mycobacterium tuberculosis reveals a cAMP-responsive RNA that is expressed during infection. Gene.

[23] DeJesus M.A., Gerrick E.R., Xu W., Park S.W., Long J.E., Boutte C.C. (2017). Comprehensive essentiality analysis of the Mycobacterium tuberculosis genome via saturating transposon mutagenesis. mBio.

[24] Comas I., Coscolla M., Luo T., Borrell S., Holt K.E., Kato-Maeda M. (2013). Out-of-Africa migration and Neolithic coexpansion of Mycobacterium tuberculosis with modern humans. Nat Genet.

[25] Griffin J.E., Gawronski J.D., DeJesus M.A., Ioerger T.R., Akerley B.J., Sassetti C.M. (2011). High-resolution phenotypic profiling defines genes essential for mycobacterial growth and cholesterol catabolism. PLoS Pathog.

[26] Auganova D., Atavliyeva S., Amirgazin A., Akisheva A., Tsepke A., Tarlykov P. (2023). Genomic characterization of drug-resistant Mycobacterium tuberculosis L2/Beijing isolates from Astana, Kazakhstan. Antibiotics.

[27] Payne J.L., Menardo F., Trauner A., Borrell S., Gygli S.M., Loiseau C. (2019). Transition bias influences the evolution of antibiotic resistance in Mycobacterium tuberculosis. PLoS Biol.

[28] Qian W., Ma N., Zeng X., Shi M., Wang M., Yang Z. (2024). Identification of novel single nucleotide variants in the drug resistance mechanism of Mycobacterium tuberculosis isolates by whole-genome analysis. BMC Genom.

[29] Liu Y., Li H., Dai D., He J., Liang Z. (2024). Gene regulatory mechanism of Mycobacterium tuberculosis during dormancy. Curr Issues Mol Biol.

[30] Mvubu N.E., Jacoby K. (2023). *Mycobacterium tuberculosis* complex molecular networks and their regulation: implications of strain heterogeneity on epigenetic diversity and transcriptome regulation. Heliyon.

[31] Chiner-Oms Á., López M.G., Moreno-Molina M., Furió V., Comas I. (2022). Gene evolutionary trajectories in Mycobacterium tuberculosis reveal temporal signs of selection. Proc Natl Acad Sci.

[32] Khademi S.M.H., Sazinas P., Jelsbak L. (2019). Within-host adaptation mediated by intergenic evolution in Pseudomonas aeruginosa. Genome Biol Evol.

[33] Worakitchanon W., Yanai H., Piboonsiri P., Miyahara R., Nedsuwan S., Imsanguan W. (2024). Comprehensive analysis of Mycobacterium tuberculosis genomes reveals genetic variations in bacterial virulence. Cell Host Microbe.

[34] Bei C., Zhu J., Culviner P.H., Gan M., Rubin E.J., Fortune S.M. (2024). Genetically encoded transcriptional plasticity underlies stress adaptation in Mycobacterium tuberculosis. Nat Commun.

[35] Comas I., Borrell S., Roetzer A., Rose G., Malla B., Kato-Maeda M. (2012). Whole-genome sequencing of rifampicin-resistant Mycobacterium tuberculosis strains identifies compensatory mutations in RNA polymerase genes. Nat Genet.

[36] Ho T.B., Robertson B.D., Taylor G.M., Shaw R.J., Young D.B. (2000). Comparison of Mycobacterium tuberculosis genomes reveals frequent deletions in a 20 kb variable region in clinical isolates. Yeast Chichester Engl.

[37] Singh A., Gaur M., Sharma V., Khanna P., Bothra A., Bhaduri A. (2021). Comparative genomic analysis of mycobacteriaceae reveals horizontal gene transfer-mediated evolution of the CRISPR-cas system in the Mycobacterium tuberculosis complex. mSystems.

[38] Napier G., Campino S., Phelan J.E., Clark T.G. (2023). Large-scale genomic analysis of Mycobacterium tuberculosis reveals extent of target and compensatory mutations linked to multi-drug resistant tuberculosis. Sci Rep.

[39] Pruthi S.S., Billows N., Thorpe J., Campino S., Phelan J.E., Mohareb F. (2024). Leveraging large-scale Mycobacterium tuberculosis whole genome sequence data to characterise drug-resistant mutations using machine learning and statistical approaches. Sci Rep.

[40] Luo Y., Huang C.-C., Howard N.C., Wang X., Liu Q., Li X. (2024). Paired analysis of host and pathogen genomes identifies determinants of human tuberculosis. Nat Commun.

[41] Tarazona D., Jaramillo L., Borda V., Levano K., Galarza M., Guio H. (2017). A genomic signature for genotyping Mycobacterium tuberculosis. Bioinformation.

[42] Wang J.-L., Chen Y.-L., Guan C.-P., Yu K., Wang M.-S. (2025). Clinical Mycobacterium tuberculosis isolates exhibit a molecular clock rate below 1 SNP per genome per year. Front Microbiol.

[43] Shukla S., Bhardwaj N., Singh A. (2024). Drug resistance in *Mycobacterium tuberculosis*: an evolutionary perspective and its adaptation to the lung microenvironment. Microbe.

[44] Farhat M.R. (2019). GWAS for quantitative resistance phenotypes in Mycobacterium tuberculosis reveals resistance genes and regulatory regions. Nat Commun.

