# Supplementary material for: Genomic landscape of Mycobacterium tuberculosis: Identifying mutation hotspots and stable regions for implications for drug development
Source: New Microbes New Infect. 2025 Nov 20;68:101674. doi: 10.1016/j.nmni.2025.101674 (PMC12686633; doi:10.1016/j.nmni.2025.101674)
Supplement: Multimedia component 2 [file mmc2.pdf]

Circos Plot R Script Documentation

Overview

This R script processes genomic mutation data and generates comprehensive visualizations using circos plots, scatter plots, frequency distribution maps, and pie charts. The analysis is performed for both the combined dataset and individual sample datasets.

Code Block Structure

1. Setup and Library Loading (Lines 1-17)

Purpose: Initialize the R environment and load required packages.

Key Operations:

- Set display precision to 22 digits for accurate numeric representation
- Load essential libraries:
  - `circlize`: For circos plot generation
  - `tidyverse`, `tidyfst`: Data manipulation and fast operations
  - `ComplexHeatmap`: Advanced visualization support
  - `ggplot2`: Statistical graphics
  - `xlsx`, `openxlsx`: Excel file I/O
  - `readr`: Data import utilities

---

2. Data Input Block (Lines 19-40)

Purpose: Load and preprocess raw mutation data files.

Key Operations:

- Set working directory to data folder
- Read all CSV files from the data directory
- Create a list structure `a` to store dataframes
- Skip first 2 rows of each CSV file (header information)
- Rename columns:
  - `Position` → `Pos`
  - `Reference` → `Ref`
  - `Sample` → `Mut`
- Convert `Pos` column to numeric format
- Add sample identifier column `sampleID` to track data origin

Input Data Format:
Expected CSV structure with columns: Position, Reference, Sample, and other metadata.

Data Structure:

```
Position | Reference | Sample | ... (other columns)
   1234  |     A     |   G    |
   5678  |     C     |   T    |
```

---

3. Data Merging Block (Lines 44-51)

Purpose: Combine all individual sample dataframes into a single merged dataset.

Key Operations:

- Initialize merge starting with first dataset `a[[1]]`
- Iteratively append remaining datasets using `rbind()`
- Result: `rstart` contains all mutation records with sample identifiers

---

4. Reference Coordinate Setup Block (Lines 53-67)

Purpose: Create coordinate reference file for circos plot initialization.

Key Operations:

- Define genome reference with:
  - Chromosome name: "All Sample"
  - Start position: 0
  - End position: max(Pos) + 10000 (added margin for edge visibility)
  - Reference base: "21B1"
  - Annotation: "n/a"
- Save as tab-delimited file in `cytoband/` directory
- File format: Name, Start, End, Reference, Annotation

---

5. Directory Creation Block (Lines 70-92)

Purpose: Establish output directory structure for results.

Key Operations:

- Create main output folder: `output2/All Sample/`
- Create individual sample folders: `output2/{sample_name}/` for each sample
- Enable file organization for downstream outputs

---

6. Combined Dataset Analysis Block (Lines 94-309)

Purpose: Analyze and visualize mutation patterns for all samples combined.

6.1 Mutation Position Statistics (Lines 94-131)

Operations:

- Replace NA values with "NULL" string
- Count mutation occurrences at each genomic position
- Create position-value dataframe for circos visualization:
  - Chromosome: "All Sample"

- Start: Position
- End: Position + 1
- Value: Mutation count

6.2 Circos Plot Generation (Lines 123-172)

Circos Plot Mathematical Framework:

The circos plot visualizes genomic coordinates in a circular layout. The mathematical transformation follows these principles:

Angular Mapping:

- Total genomic span: [0, max(Position)]
- Circular circumference: 360° divided by sectors
- Gap between sectors: 20° (configurable via `gap.after`)
- Start angle: 87.5° (clockwise from vertical)

Track Layout (from center outward):

1. **Track 1 - Chromosome Label:** Display "All Sample" text at sector center
    - Radial position: ylim center
    - Angular position: sector mean angle
    - Transformation: (x, y) → (θ, r) where θ is arc angle, r is distance from center
2. **Track 2 - Genomic Points:** Red dots representing mutations
    - Height represents mutation count at position
    - Position: (angle corresponding to genomic position, radial height = mutation count)
    - Formula:

    ```
    θ = (Pos / max(Pos)) × (360° - total_gaps)
    r = value / max(value)
    ```

3. **Track 3 - Kernel Density Estimation:** Black line showing mutation density
    - Kernel bandwidth determined by automatic calculation
    - Smooths discrete point data for pattern visualization
    - Density function: $d(x) = (1/nh) \times \Sigma K((Pos_i - x)/h)$
      where K is the kernel function, h is bandwidth

Parameters:

- `circos.par(gap.after = 20)`: Sector gap in degrees
- `start.degree = 87.5`: Starting angle
- `gap.degree = 3`: Additional spacing
- Color scheme: Grey background for tracks, red for mutation points

Plot Output: PDF file (10×10 inches) at `output2/All Sample/All Sample_circos_plot.pdf`

6.3 Statistical Plots (Lines 204-229)

Scatter Plot:

- X-axis: Genomic position
- Y-axis: Number of mutations at position
- Visual type: Red points
- Purpose: Identify hotspot regions

Frequency Distribution Histogram:

- Bin width: (max(Pos) - min(Pos)) / 500
- Shows distribution of mutations across genome
- Identifies clusters and sparse regions

Outputs:

- `{sample}/Scatter plot.png`
- `{sample}/Frequency Diagram.png`

6.4 Mutation Type Summary (Lines 232-309)

Purpose: Analyze base substitution patterns.

Operations:

- Combine Reference and Mutation bases: `Ref/Mut` (e.g., "A/G")
- Count frequency of each substitution type
- Calculate proportion: sequence = count / total_count
- Filter: Group minor variants (< 1% frequency) as "other"

Pie Chart Generation:

- 2D Pie Chart: Color-coded by mutation type with percentages
- 3D Pie Chart: Exploded view for emphasis
- Output files:
    - `All Sample/the 2D pie of base mutation.png`
    - `All Sample/the 3D pie of base mutation.png`

---

7. Individual Sample Analysis Loop (Lines 320-407)

Purpose: Perform detailed analysis for each individual sample.

Structure: `for(iw in names(a))` iterating through each sample

Operations Per Sample:

1. Extract individual sample data from list `a[[iw]]`
2. Replicate steps 6.1-6.4 for single sample:
    - Position-based mutation statistics
    - Base substitution type analysis
    - Proportion calculations
3. Export results to Excel:
    - File: `output2/{sample}/{sample}.xlsx` - Raw mutation data with combined bases
    - File: `output2/{sample}/coundut of {sample}.xlsx` - Summary statistics with:
        - Sheet 1: Position counts
        - Sheet 2: Base substitution totals and proportions
        - Sheet 3: Position-specific mutation types
4. Generate visualizations:
    - 2D Pie Chart: `output2/{sample}/the 2D pie of base mutation of {sample}.png`
    - 3D Pie Chart: `output2/{sample}/the 3D pie of base mutation of {sample}.png`

Data Flow Summary

```
CSV Input Files
       ↓
   [Read & Preprocess]
       ↓
   List of Dataframes (a)
       ↓
   [Merge into rstart]
       ↓
   ├─→ [All Sample Analysis]
   │       ├─→ Circos Plot (PDF)
   │       ├─→ Scatter Plot (PNG)
   │       ├─→ Frequency Distribution (PNG)
   │       └─→ Pie Charts (PNG × 2)
   │
   └─→ [Individual Sample Loop]
           ├─→ Statistics Excel files
           └─→ Pie Charts (PNG × 2) per sample
```

Mathematical Details

Circos Plot Coordinate System

Polar to Cartesian Conversion:
For each mutation at position Pos with count value:

1. **Angular Position:**
   - Position normalized: pos_norm = Pos / max(Pos)
   - Sector angle range: [start.degree, start.degree + (360 - total_gaps)]
   - Point angle: θ = start.degree + pos_norm × (360 - total_gaps)
2. **Radial Position:**
   - Value normalized: value_norm = value / max(value)
   - Radial distance: r = r_track_base + value_norm × track_height
   - r_track_base depends on track number (inner, middle, outer)
3. **Cartesian Coordinates:**
   - x = r × cos(θ)
   - y = r × sin(θ)

Kernel Density Estimation in Track 3

KDE Formula:

```
f(x) = (1/(n×h)) × Σ(i=1 to n) K((Pos_i - x)/h)
```

Where:

- n: number of mutation points
- h: bandwidth parameter (automatically selected)
- K: kernel function (typically Gaussian)
- Pos_i: individual mutation positions

Gaussian Kernel:

```
K(u) = (1/√(2π)) × exp(-u²/2)
```

This smooths discrete point distributions into continuous density curves for pattern identification.

Proportion Calculation

Base Substitution Proportions:

```
sequence[i] = count[i] / Σ(all counts)
```

Where count[i] is the frequency of substitution type i.

Filtering Rule:

- If sequence[i] < 0.01 (1%), grouped into "other" category
- New "other" count = Σ(counts where sequence < 0.01)

Output Summary

For Combined Dataset (All Samples)

1. **PDF:** Circos plot with mutation visualization
2. **PNG (2):** Scatter and frequency distribution plots
3. **PNG (2):** 2D and 3D pie charts of base substitutions
4. **XLSX:** Summary statistics and mutation details

For Each Individual Sample

1. **PNG (2):** 2D and 3D pie charts specific to sample
2. **XLSX:** Sample-specific mutation data and statistics

File Organization

```
output2/
├── All Sample/
|   ├── All Sample_circos_plot.pdf
|   ├── All Sample_Scatter plot.png
|   ├── All Sample_Frequency Diagram.png
|   ├── the 2D pie of base mutation.png
|   ├── the 3D pie of base mutation.png
|   └── (Excel files)
|
└── {sample_name}/
    ├── the 2D pie of base mutation of {sample_name}.png
    ├── the 3D pie of base mutation of {sample_name}.png
    ├── {sample_name}.xlsx
    └── coundut of {sample_name}.xlsx
```

Dependencies

Required R packages: `circlize`, `tidyverse`, `tidyfst`, `ComplexHeatmap`, `ggplot2`, `xlsx`, `openxlsx`, `readr`, `plotrix`, `ggpubr`

Ensure all packages are installed before running the script.
